# Resting energy expenditure in elite athletes: development of new predictive equations based on anthropometric variables and bioelectrical impedance analysis derived phase angle

**DOI:** 10.1186/s12970-021-00465-x

**Published:** 2021-10-26

**Authors:** Maurizio Marra, Olivia Di Vincenzo, Iolanda Cioffi, Rosa Sammarco, Delia Morlino, Luca Scalfi

**Affiliations:** 1 Department of Clinical Medicine and Surgery, Federico II University of Naples, Via S. Pansini 5, 80138 Naples, Italy; 2 Department of Public Health, Federico II University of Naples, Via S. Pansini 5, 80138 Naples, Italy

**Keywords:** Athletes, Energy expenditure, Predictive equations, BIA, Phase angle, Accuracy

## Abstract

**Background:**

An accurate estimation of athletes’ energy needs is crucial in diet planning to improve sport performance and to maintain an appropriate body composition. This study aimed to develop and validate in elite athletes new equations for estimating resting energy expenditure (REE) based on anthropometric parameters as well as bioimpedance analysis (BIA)-derived raw variables and to validate the accuracy of selected predictive equations.

**Methods:**

Adult elite athletes aged 18–40 yrs were studied. Anthropometry, indirect calorimetry and BIA were performed in all subjects. The new predictive equations were generated using different regression models. The accuracy of the new equations was assessed at the group level (bias) and at the individual level (precision accuracy), and then compared with the one of five equations used in the general population or three athletes-specific formulas.

**Results:**

One-hundred and twenty-six male athletes (age 26.9 ± 9.1 yrs; weight 71.3 ± 10.9 kg; BMI 22.8 ± 2.7 kg/m^2^) from different sport specialties were randomly assigned to the calibration (*n* = 75) or validation group (*n* = 51). REE was directly correlated with individual characteristics, except for age, and raw BIA variables. Most of the equations from the literature were reasonably accurate at the population level (bias within ±5%). The new equations showed a mean bias −0.3% (Eq. A based on anthropometric parameters) and −0.6% (Eq. B based on BIA-derived raw variables). Precision accuracy (individual predicted-measured differences within ±5%) was ~75% in six out of eight of the selected equations and even higher for Eq. A (82.4%) and Eq. B (92.2%).

**Conclusion:**

In elite athletes, BIA-derived phase angle is a significant predictor of REE. The new equations have a very good prediction accuracy at both group and individual levels. The use of phase angle as predictor of REE requires further research with respect to different sport specialties, training programs and training level.

## Background

Total energy expenditure of most athletes is expected to be greater compared to general population because of training, and changes in metabolism and body composition [1]. At the same time, estimating energy needs is crucial in diet planning to improve sport performance and manage body mass in weight-category sports [2, 3]. Further, under- or over-estimating athletes’ energy requirements might result in unwanted changes in fat-free mass (FFM), and/or fat mass (FM), impaired performance and health concerns, for instance increased risk of injuries or cardiovascular diseases [1, 2, 4].

Energy requirements may be assessed based on resting energy expenditure (REE) [5], which is the amount of energy expended at rest by a fasted individual in a thermoneutral environment, representing 60–70% of total energy expenditure in normal-weight healthy adults and variable percentages in athletes [6]. In human nutrition REE is commonly estimated by using predictive equations based on easily available variables such as age, stature, body weight, etc. Most of the widely used equations for estimating REE in the general population (Harris and Benedict (HB) [7], Schofield [8], FAO/WHO/UNU [9], Mifflin [10] and Owen [11]) have been developed based on minimally active or sedentary individuals. Taking into account different physical activity level and body composition (i.e. higher FFM and body cell mass with lower FM) relative to general population [12–14], the equation used for estimating REE in the general population may be not appropriate for athletic individuals. As a consequence, few specific predictive equations for REE have been developed for athletes, [15–18], indeed exhibiting some limitations. The study by De Lorenzo et al. included a small sample size (*n* = 51) [15] whereas the one by ten Haaf et al. involved recreational athletes exercising on average 9.1 ± 5 h a week [17]. Wong et al. studied Asiatic athletes only [16] and Watson et al. [18] only females. Finally, Jagim et al. [5] determined the accuracy of selected predictive equations for REE, but they did not propose a validated formula.

REE is expected to be associated with FFM, which is a reasonable surrogate body composition marker for oxidatively active tissues [19]; in other word, FFM might be used for predicting REE, and from a practical point of view this is even more credible if field techniques are employed. In this perspective, bioelectrical impedance analysis (BIA) is widely used for assessing body composition in athletes [20], but the interpretation of BIA results depends to a large extent on the equation used to estimate FFM [21]. Interestingly, raw BIA variables such as bioimpedance index (BI-Index = stature^2^/impedance at 50 kHz) and phase angle (PhA) might be taken into account as possible predictors of REE. In fact, while BI-index is strictly related to FFM, PhA is thought to be a proxy of both water distribution (i.e. the ratio between extracellular water-ECW and total body water-TBW) [22], body cell mass and cellular integrity [23]. So far, a relationship between REE and BI-index and/or PhA has already been observed in normal-weight or overweight subjects [24] as well as in patients with obesity [25] and Crohn’s disease [26]. Not surprisingly, the results of these studies suggest that raw BIA variables may improve the prediction power under physiological conditions [24], but only to a limited extent in subjects with altered body water distribution [25, 26].

Based on this background, the primary aim of this study was to develop and validate new predictive equations of REE in elite athletes, considering not only anthropometric measures, but also raw BIA variables. The accuracy of new equations, as well as the one of selected predictive equations of REE used in the general population or in athletes, was evaluated at the group level (bias) and at the individual level (precision).

## Methods

### Study design and subjects

In the present study we have retrospectively analysed routine data collected between January 2012 and December 2019 in elite athletes defined as those who have previously competed as regional and/or national players [27].

Subjects were selected for this study according to the following inclusion criteria: (1) both sexes, (2) age between 18 and 45 yrs and (3) at least 24 h/week of training. Subjects affected by overt metabolic and/or endocrine diseases and/or regularly taking any medications or using any drugs affecting energy metabolism, were excluded. This study was conducted in accordance with the Declaration of Helsinki and was approved by the Federico II University Ethical Committee.

All measurements were performed early in the morning (8.30 a.m.) after an overnight fast (10–12 h) according to standardized conditions, i.e. abstention from alcohol, caffeine or other thermogenetic substances, smoking and any physical activity for 24 h (in most cases 36 h) prior to the assessment.

### Anthropometry and bioelectrical impedance analysis

Body weight was measured in duplicate to the nearest 0.1 kg using a platform beam scale and stature was measured in triplicate to the nearest 0.5 cm using a stadiometer (Seca 709; Seca Hamburg, Germany). The subject wore light clothes and no shoes. Body mass index (BMI) was calculated as body weight (kg) divided by squared stature (m^2^).

BIA was performed by phase-sensitive device (Human IM Touch, DS Medica S.r.l., Milan, Italy). Measurements were carried out with empty bladder, in a supine position for at least 10 min before starting the measurement). After cleaning skin surface, patients were asked to lay with upper and lower limbs slightly abducted, so there was no contact between the extremities and trunk. The measuring electrodes were placed on the anterior surface of the wrists and ankles, and the injecting electrodes were placed on the dorsal surface of the hands and the feet, respectively (overall, eight electrodes). Data for impedance and PhA from the non-dominant side of the body, measured at 50 kHz, were considered. BI-index was calculated as the ratio stature^2^/resistance (cm^2^/ohm).

Before each test the analyser was calibrated with the calibration considered successful if resistance value was between 382 and 385 Ω and reactance was 44–46 Ω, as indicated by the manufacturer guidelines. The test-retest coefficient of variation (CV) (as determined in ten subjects) was always less than 3%.

### Resting energy expenditure

REE was measured (MREE) by indirect calorimetry [28] using a canopy system (Vmax® Encore system, CareFusion Corporation, U.S.). The instrument was routinely checked by burning ethanol, whereas oxygen and carbon dioxide analysers were calibrated on the test day using nitrogen and standardized gases (mixtures of nitrogen, carbon dioxide and oxygen).

Measurement conditions for by indirect calorimetry were defined following the suggestions made by Compher et al. [29] and Fullmer et al. [30]. In addition to the standardized conditions already mentioned, REE was measured with the subject laying down, but awake, on a bed in a quiet environment. After a 15-min adaptation period, oxygen consumption and carbon dioxide production were measured for 45 min. Only steady state periods of measurement were selected according to the procedures for the ventilated hood system (< 5% CV). The first 5 min were discarded. Also, the inter-day CV (as determined in 10 subjects on subsequent days) was always less than 4%. The flow throughout the canopy was modified in order to maintain the CO_2_ between 0.6–0.8%.

Energy expenditure was calculated using the abbreviated Weir’s formula, neglecting protein oxidation [31]. Data were excluded from analysis if the respiratory quotient was outside the expected range (0.71–1.00) and when measured REE was ±3 standard deviations outside the mean REE.

### Predictive equations

In the validation group REE was predicted (PREE) using five equations that are widely mentioned with respect to the general population (Harris & Benedict [7], Schofield [8], FAO/WHO/UNU [9], Mifflin [10] and Owen [11]), and three athletes-specific formulas from the literature (De Lorenzo [15], Wong [16] and ten Haaf [17])(Table 1).

**Table 1:** Resting energy expenditure predictive equations in their original unit (kcal/day, except Schofield and FAO/WHO/UNU (MJ/day))

Equation	Formula
Harris and Benedict [7]	Males 13.75 × Weight (kg) + 5 × Stature (cm) – 6.76 × Age (yrs) + 66.47
Schofield [8]	Males (18–30 yrs) 63 × Weight (kg) – 42 × Stature (m) + 2953 Males (30–60 yrs) 48 × Weight (kg) – 11 × Stature (m) + 3670
FAO/WHO/UNU [9]	Males (18–30 yrs) 15.3 × Weight (kg) – 27 × Stature (m) + 679 Males (30–60 yrs) 11.6 × Weight (kg) – 16 × Stature (m) + 879
Mifflin [10]	9.99 × Weight (kg) + 6.25 × Stature (cm) – 4.92 × Age (yrs) + 166 × Sex (M = 1, F = 0) – 161
Owen [11]	Males 10.2 × Weight (kg) + 879
De Lorenzo [15]	9 × Weight (kg) + 11.7 × Stature (cm) − 857
Wong [16]	13 × Weight (kg) + 192 × Sex (M = 1, F = 0) + 669
Ten Haaf [17]	11.936 × Weight (kg) + 587.728 × Stature (cm) – 8.129 × Age (yrs) + 191.027 × Sex (M = 1, F = 0) + 29.279

### Statistical analysis

Statistical analyses were performed using IBM SPSS (version 26). All data are presented as mean ± standard deviation (SD), unless otherwise specified, and significance was defined as *p* < 0.05. The Kolmogorov-Smirnov Test and the Shapiro-Wilk Test were used to assess if variables were normally distributed.

As presented in Table 2, subjects were randomly assigned to either a calibration or a validation group.

**Table 2:** Characteristics of the study sample for the calibration and validation groups

	All (***n*** = 126)	Calibration group (***n*** = 75)	Validation group (***n*** = 51)
Age, yrs	26.9 ± 9.1	26.8 ± 9.0	27.1 ± 9.5
Weight, kg	71.3 ± 10.9	71.4 ± 11.3	71.1 ± 10.6
Stature, cm	177 ± 7	177 ± 7	177 ± 7
BMI, kg/m^2^	22.8 ± 2.7	22.8 ± 2.8	22.8 ± 2.6
MREE, kcal/die	1831 ± 250	1834 ± 261	1826 ± 234
RQ	0.821 ± 0.07	0.832 ± 0.07	0.822 ± 0.07
BI-index, cm^2^/Ω	65.9 ± 9.6	65.9 ± 9.8	66.0 ± 9.4
PhA, degrees	7.76 ± 0.76	7.79 ± 0.74	7.73 ± 0.78

Data are expressed as mean ± standard deviation

*BMI* body mass index, *MREE* measured resting energy expenditure, *RQ* respiratory quotient, *BI-index* bioimpedance index, *PhA* phase angle

As far as statistical power is concerned, in the calibration group for alpha level = 0.05 and beta = 0.20 a sample size of 75 subjects is requested to reach a *p* < 0.05 for *r* = 0.330 (*R*^2^ = 0.10). In the validation group a sample size of 51 subjects is adequate to identify a significant between-groups difference of 50 kcal with a standard deviation of 125 kcal.

Linear correlation was applied for evaluating associations between variables. Multivariate linear regression analysis was performed to develop the new predictive equations, with REE measured by indirect calorimetry as dependent variable. We generated models as follows: in Model 1, age, sex, weight, stature and BMI were set as predictors, while in Model 2 we added the raw BIA variables (BI-index and PhA). Coefficient of determination (R^2^) and standard error of the estimate (SEE) were considered for assessing the predictive power of formulas. The regression equations, derived from the calibration subset, were applied to the validation group.

Differences between PREE and MREE as well as bias, i.e. the mean percent difference, were both used as a measure of accuracy at the population level. Bias was found acceptable if within ±5% [32, 33]. The percentage of patients with a PREE within 90–110% of MREE was used as a measure of accuracy at the individual level (precision accuracy). Values lower than 90% were classified as underprediction, while values higher than 110% as overprediction. The root mean squared error (RMSE) was used to define the predictions obtained with these models. Finally, comparisons of PREE-MREE differences vs mean PREE-MREE values were performed by Bland and Altman plots to estimate the limits of agreement [34].

## Results

One hundred and twenty-six male elite athletes from different sport specialties were included in the analysis. As mentioned above, data on anthropometric measures, raw BIA variables and MREE are reported for the calibration and validation groups in Table 2. Athletes from seven sports were recruited, practicing masters swimming (*n* = 24, 19%), cycling (*n* = 22, 17.5%), running (*n* = 21, 16.7%), karate (*n* = 17, 13.5%), water polo (*n* = 16, 12.7%), ballet dance (*n* = 15, 11.9%) and boxing (*n* = 11, 8.7%). Individual characteristics for each sport specialty are reported in Table 3. BMI was the highest in water polo players (25.9 ± 1.8 kg/m^2^) and the lowest in runners (20.6 ± 1.2 kg/m^2^). MREE was the highest in water polo players (2195 ± 244 kg/day) and the lowest in ballet dancers (1567 ± 107 kg/day) in line with the differences in body weight. Mean value of PhA varied between 8.57 ± 0.65 degrees in boxers and 6.96 ± 0.54 degrees in master swimmers, being higher in boxers, cyclists and water polo players (Table 3).

**Table 3:** Characteristics of the study sample according to sport specialty

	Cycling	Water polo	Masters swimming	Karate	Ballet Dance	Boxing	Running
	(*n* = 22)	(*n* = 16)	(*n* = 24)	(*n* = 17)	(*n* = 15)	(*n* = 11)	(*n* = 21)
Age (yrs)	27.0 ± 2.7^cdef^	24.2 ± 6.6^c^	40.4 ± 4.5^abdefg^	18.8 ± 2.7^aceg^	19.1 ± 1.1^acg^	20.7 ± 2.7^aeg^	29.0 ± 9.9^cdf^
Weight (kg)	69.2 ± 5.2^bcg^	88.8 ± 4.9^acdefg^	76.6 ± 10.0^abdeg^	69.5 ± 10.4^bcg^	64.1 ± 5.2^bc^	70.0 ± 5.2^bg^	61.1 ± 6.3^abcdg^
Stature (cm)	181 ± 6^defg^	185 ± 3^cdefg^	176 ± 5^bf^	176 ± 7^ab^	175 ± 4^ab^	169 ± 5^abc^	172 ± 5^ab^
BMI (kg/m^2^)	21.2 ± 1.3^bcf^	25.9 ± 1.8^adeg^	24.6 ± 2.8^adeg^	22.5 ± 2.7^bcg^	20.9 ± 0.9^bcf^	24.7 ± 0.6^aeg^	20.6 ± 1.2^bcdf^
MREE (kcal/die)	1866 ± 142^beg^	2195 ± 244^acdefg^	1766 ± 188^bde^	1928 ± 207^bceg^	1567 ± 107^abcdf^	1946 ± 127^beg^	1641 ± 120^abdf^
RQ	0.785 ± 0.031^beg^	0.865 ± 0.046^a^	0.815 ± 0.081	0.804 ± 0.049^g^	0.850 ± 0.049^a^	0.807 ± 0.082	0.870 ± 0.070^ad^
BI-index (cm^2^/Ω)	65.4 ± 8.0^bg^	78.8 ± 8.3^acdefg^	67.6 ± 7.7^bg^	64.0 ± 8.7^bg^	67.7 ± 6.3^bg^	65.9 ± 5.7^bg^	55.2 ± 5.0^abcdef^
PhA (degrees)	8.31 ± 0.79^cdg^	8.11 ± 0.49^c^	6.96 ± 0.54^abdefg^	7.59 ± 0.60^acf^	7.75 ± 0.53^cf^	8.57 ± 0.65^cdeg^	7.60 ± 0.38^acf^

Data are expressed as mean ± standard deviation.

*BMI* body mass index, *MREE* measured resting energy expenditure, *RQ* respiratory quotient, *BI-index* bioimpedance index, *PhA* phase angle.

^a^cycling; ^b^ water polo; ^c^ master swimming; ^d^ karate; ^e^ ballet dance; ^f^ boxing; ^g^ running

*p* < 0.05

### Developing new predictive equations

Linear correlations showed that MREE of the athletes directly correlated with individual characteristics and raw BIA variables, except for age (*r* = − 0.124, *p* = 0.290). Actually, a strong correlation was found between MREE and body weight (*r* = 0.768, *p* < 0.001), followed by BMI (*r* = 0.623, *p <* 0.001), BI-index (*r* = 0.606, *p <* 0.001) as an index of FFM, stature (*r* = 0.489, *p <* 0.001) and PhA (*r* = 0.327, *p* = 0.004).

Then, multiple regression analysis was performed to assess the relationship between MREE and different sets of potential predictors. Basic anthropometric measures (weight, stature and BMI) and age (although not significant in bivariate analysis) were considered first in Model 1 to generate the following Eq. A:

**Table:** 

REE (kcal/day) =	17.2 × Weight (kg) (1.5) *0.794*	− 5.95 × Age (yrs) (1.9) *− 0.218*	+ 748 (117.9)

(unstandardized regression coefficients with SE in brackets and beta coefficients in italics)

*R*^2^ = 0.637; SEE = 150 kcal/day.

When raw BIA variables (BI-index and PhA) were added to the Model 2, PhA was included whereas age was excluded from the model, developing the following Eq. B:

**Table:** 

REE (kcal/day) =	16.3 × Weight (kg) (1.5) *0.755*	+ 95.4 × PhA (degrees) (22) *0.291*	*− 93* (197)

(unstandardized regression coefficients with SE in brackets, and beta coefficients in italics)

*R*^2^ = 0.675; SEE = 141 kcal/d.

### Validation of predictive equations

To assess the accuracy of the new predictive equations, as well as of those selected from the literature, 51 athletes were randomly assigned to the validation group. Prediction accuracy at the population level was evaluated by PREE-MREE difference, mean bias and RMSE in kcal/day (Table 4).

**Table 4:** Evaluation of new and selected predictive equations in athletes (validation group)

REE predictive equations	Difference PREE-MREE kcal/d Mean (SD)	Bias^§,^ %	RMSE kcal/d
*Equations for normal-weight subjects*			
HB	− 82 (146)*	− 3.9	107
Schofield	− 93 (142)*	− 4.4	108
FAO/WHO/UNU	− 92 (140)*	− 4.4	107
Mifflin	− 141 (156)*	− 7.0	164
Owen	− 222 (140)*	− 11.3	225
*Equations for athletes*			
De Lorenzo	21 (173)	2	94
Wong	− 41 (153)*	−1.4	104
Ten Haaf	60 (152)*	4	98
Equation A	− 17 (134)	− 0.3	88
Equation B	− 20 (124)	− 0.6	76

Average REE measured with indirect calorimetry = 1826 ± 234 kcal/d

*REE* resting energy expenditure, *MREE* measured resting energy expenditure, *PREE* predicted resting energy expenditure, *RMSE* root mean square error, *HB* Harris and Benedict, *FAO* Food and Agriculture Organization

^§^ Mean percentage error between predicted and measured REE; * *p* < 0.05

**Fig. 1:**
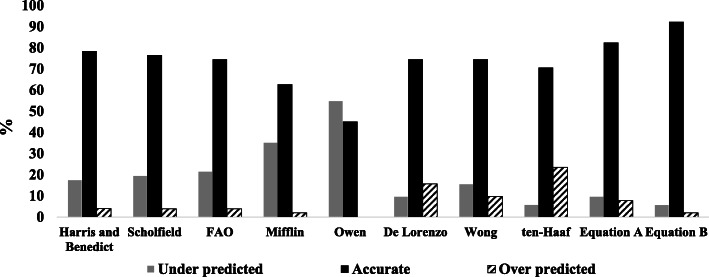
Accuracy of prediction equations for measurements of resting energy expenditure within ±10%

The new developed predictive formulas showed a mean bias < 1% (Eq. A − 0.3%; Eq. B − 0.6%) with the lowest RMSE values of 88 kcal (Eq. A) and 76 kcal (Eq. B); while REE seemed to be underestimated by most of the other equations, with the exception of those by De Lorenzo and ten Haaf (Table 4). Overall, the PREE-MREE difference was < 100 kcal/day for the HB, FAO, Schofield, De Lorenzo, Wong and ten Haaf equations. The mean bias was as follows: HB − 3.9%; Schofield + 4.4%; FAO − 4.4%; De Lorenzo + 2%; Wong − 1.4% and ten Haaf + 4%), and greater for the Mifflin (− 7%) and Owen (− 11.3%) equations.

(As shown in Fig. 1) the precision accuracy at the individual level (percentage of athletes with a PREE within ±10% of MREE) was higher for the new equations (Eq. A 82.4%, Eq. B 92.2%) compared to those selected from the literature (~ 45% for the Owen, ~ 65% for the Mifflin and ~ 75% for the Harris-Benedict, FAO, Schofield, De Lorenzo, Wong and ten Haaf equations).

### Bland-Altman plots of PREE-MREE differences

**Fig. 2:**
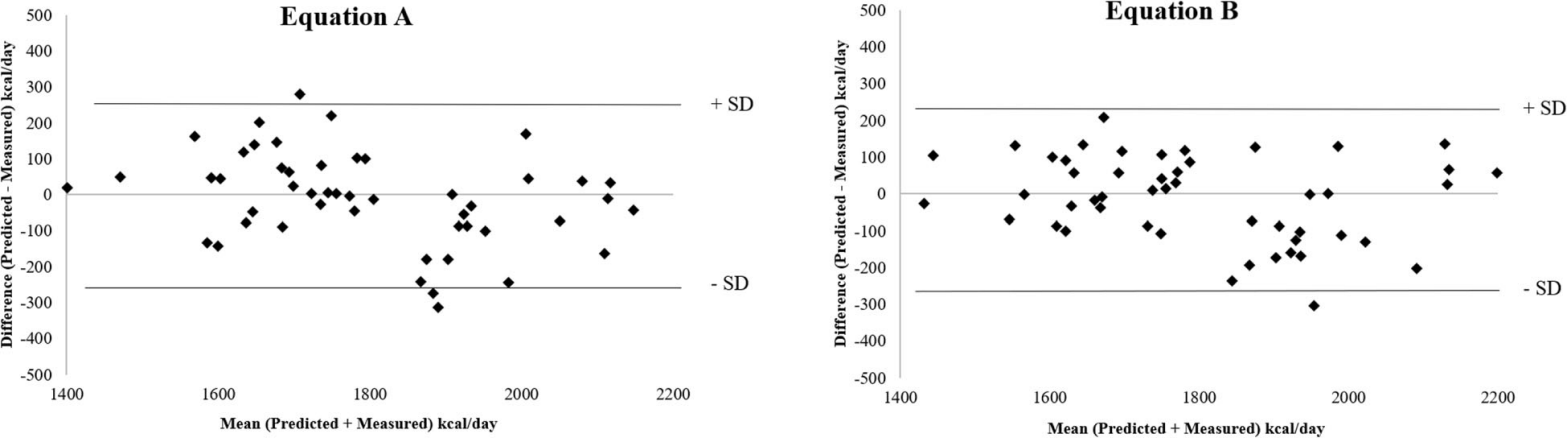
Bland - Altman plots between differences and mean predicted-measured resting energy expenditure using new equations

Lastly, the Bland-Altman method was used to quantify the agreement between PREE and MREE. Figure 2 shows that the best agreement was found for the new formulas. For the other equations, the 95% limits of agreement were wider (+/− 200–300 kcal/d) with the largest values observed for the Mifflin and Owen equations.

## Discussion

The primary purpose of this study was to develop and cross-validate new equations for estimating REE in a group of elite male athletes of different sport specialties, and then to compare them with existing formulas. The new equations provide the best prediction of REE in the validation group, with the use of BIA-derived PhA significantly improving the prediction power of the equation.

Meeting energy requirements is a priority of athletes. Inadequate energy intake might compromises performance and reduces the benefits of training [1, 2]. Energy needs are usually estimated by REE multiplied by the appropriate activity factor. To date, only a few number of predictive equations for REE have been specifically developed for athletes [15–18]. De Lorenzo formula [15] was derived in a sample of 51 male athletes (22 water polo, 12 judo, 17 karate) who exercised at least 3 h/day; in that paper REE was underestimated by most of the seven equations selected from the literature. Later, Wong et al. [16] proposed sex-specific predictive formulas for elite Malaysian athletes in most cases practicing combat sports. Of note, Malaysian population seemed to have relatively low body frames and size and, therefore, low REE [16]. They found that mean resting energy expenditure measured by indirect calorimetry were similar in males to values predicted using the HB [7], FAO [9] and De Lorenzo [15] equations; indeed the accuracy of the predictive formulas was not evaluated. Also, ten Haaf et al. [17] developed two predictive equations for recreational athletes practicing > 3 h/day two times a week, the first formula being based on weight, the second one on FFM (determined using pletismography). Authors pointed out that the weight-based equation had a higher precision accuracy (83% for males) compared to the De Lorenzo formula (77.4% for males). Finally, Watson et al. [18] derived two formulas in a sample of 66 collegiate female athletes from eleven different sports; the first equation was based on weight, the second one on FFM (estimated by skinfold thickness). Authors stated that both equations were more accurate for resting metabolic rate estimation in their population but did not evaluate bias or precision accuracy. Jagim et al. [5] did not derive new formulas but determined the accuracy of several predictive equations for REE in both male and female athletes; most of the five equations selected from the literature underestimated REE in both sexes. Of the previous studies, only the one by Watson et al. [18] described the relationships between REE and age or different anthropometric variables, showing that age was not related to REE while the best predictor was body weight (*r* = 0.590). These results are confirmed in our study since body weight was the best predictor (*r* = 0.768) while there was no association with age. Some authors also introduced FFM as predictor, with no increase in the prediction power [17, 18].

In the present study, first we developed an equation based on age and main anthropometric variables (weight, stature, and BMI) (Model 1, Eq. A). In addition to age, weight emerged as the only significant predictor. Two of the existing formulas for athletes identified also stature as predictor [15, 17] while in the athletes we studied, REE was correlated to stature in univariate analysis, but not in multiple regression analysis, *p* = 0.374).

Instead of using BIA-derived body composition (strictly dependent on the BIA formula used), we opted for including raw BIA variables (BI-index and PhA) in the regression model (Model 2, Eq. B).

BI-index is directly related to FFM and quite always included as predictor in the BIA equations to predict FFM. More recently, attention has been focused on the role of PhA as a biomarker of body cell mass and muscle quality as well as of water distribution (ratio between extracellular water-ECW and intracellular water-ICW) [22]. Thus, high PhA indicates greater cellularity (e.g. more body cell mass relative to FFM), cellular integrity and cell functions [22]. It may represent a proxy parameter of muscle quality in athletes, being significantly associated with physical activity and muscle strength [35, 36]. A recent systematic review showed that PhA was higher in athletes vs controls whereas it was still uncertain to what extent PhA differs among various sports [37]. In addition, PhA may help in detecting low muscle quality and identifying sarcopenia [38]. In previous studies, we also found that both BI-index and PhA improved the prediction power of REE under physiological conditions [24]. The findings of the present paper confirmed that PhA was as a significant predictor along with weight, with R^2^ increasing from 0.637 to 0.675 and SEE decreasing from 150 to 141 kcal/day. On the contrary, BI-index was not recognized as a stronger predictor than weight, possibly because of low body fat percentage and low BMI. In general, for those with no access to BIA, only age and weight values are sufficient for predicting REE in male elite athletes.

As additional aim, we validated the two new equations and eight formulas selected from the literature (5 for the general population and 3 for athletes), at both population and individual level. On the average, the accuracy was very good for our new formulas, since bias ranged within ±1%. Similarly, most of the selected equations, except the Mifflin and Owen ones, showed an acceptable prediction accuracy (bias ±5%).

From a practical point of view, evaluating the accuracy of predictive equations at individual level (within ±10%) is crucial for the nutritional management of the single athlete. This study shows that precision was high for the new formulas, especially for Eq. B (~ 92%) including PhA in the model while it was lower, being close to 75%, for most of the other formulas (with the exception of the Mifflin and Owen ones for which it was much lower). Looking at the Bland-Altman plots, most of the prediction equations were more accurate at lower ranges of MREE and less accurate with the higher REE values. The new formulas gave the narrowest limits of agreement and the lowest bias.

To the best of authors’ knowledge, this is the first study that developed and cross-validate equations for elite athletes to predict REE based not only on anthropometric measures, but also on raw BIA variables. Overall, we conducted this study in a reasonable large sample of individuals, using recognized and well-documented methods and in line with similar previous studies in healthy subjects. Furthermore, the assessment of BIA with the same device has limited the device-related changes in PhA. Nevertheless, these findings are subject by a number of limitations. Since this is a retrospective study, our findings need to be confirmed in larger samples and in different sports disciplines. Additionally, we studied elite athletes mostly practicing endurance sports. Lastly, female athletes were excluded from the analysis due to the small number of potential participants (*n* = 27); therefore, we have developed new athlete-specific predictive equations for estimating REE in elite male athletes only.

## Conclusions

As main finding, in elite athletes BIA-derived PhA is a significant predictor of REE and improved the prediction power of the model. The new equations exhibited a very good accuracy at population level, while precision at the individual level was markedly higher compared to that reported by previous studies in the general population as well as athletes. However, the use of PhA as predictor of REE requires further research with respect to different sport specialties, training programs and training level.

## Data Availability

All data pertaining to the conclusions of the study are found within the article. The corresponding data set used is available under reasonable requests.

## Abbreviations

BI-Index: Bioimpedance Index
BIA: Bioelectrical Impedance Analysis
BMI: Body Mass Index
ECW: Extracellular Water
FAO: Food and Agriculture Organization
FFM: Fat-Free Mass
FM: Fat Mass
HB: Harris and Benedict
ICW: Intracellular Water
MREE: Measured Resting Energy Expenditure
PhA: Phase Angle
PREE: Predicted Resting Energy Expenditure
REE: Resting Energy Expenditure
TBW: Total Body Water
